# Post-saphenous vein ablation compression therapy practices: a study among members of the Brazilian society of angiology and vascular surgery

**DOI:** 10.1590/1677-5449.202301822

**Published:** 2025-02-14

**Authors:** Fabricio Duarte, Flavia Del Castanhel, Marcondes Antônio de Medeiros Figueiredo, Getúlio Rodrigues de Oliveira

**Affiliations:** 1 Universidade Federal de Santa Catarina – UFSC, Florianópolis, SC, Brasil.; 2 Centro Universitário IMEPAC, Araguari, MG, Brasil.

**Keywords:** venous insufficiency, varicose veins, compression stockings, compression bandages, saphenous vein, ablation techniques

## Abstract

**Background:**

Despite considerable study, there is still no consensus defining the ideal compression regimen after ablation of the great saphenous vein with radio frequency or endolaser.

**Objective:**

To identify the Brazilian Society of Angiology and Vascular Surgery members’ current compression therapy practices after ablation of the great saphenous vein.

**Methods:**

A multiple-choice electronic questionnaire on post-endovenous ablation compression was developed and made available on-line to Brazilian vascular surgeons for 60 days.

**Results:**

A total of 430 responses were received, 362 (84.2%) of which were considered valid. Laser ablation was the predominant technique (73.5%) and the majority of procedures were conducted in hospitals or day hospitals. Ninety-four percent of the surgeons treated associated varicose veins in the same procedure, for which phlebectomy was the technique most employed. After ablation of the great saphenous vein, 99% of the surgeons applied compression immediately; 34.3% used 35 mmHg compression stockings, 26% preferred crepe bandages, and 12.4% opted for 20-30 mmHg compression stockings, with an average duration of 2.79 (±2 days). After this period, 88.4% used additional compression, with 20-30 mmHg compression stockings (80.9%) and an average duration of 39.3 (±24.0 days).

**Conclusions:**

Compression therapy is widely employed after thermal ablation of the great saphenous vein. Practice immediately after ablation was divergent, but after the initial phase, the majority of surgeons prescribed additional compression, predominantly using 20-30 mmHg stockings.

## INTRODUCTION

Great saphenous vein (GSV) incompetence is the most common cause of chronic venous insufficiency. In Brazil, the prevalence of lower limb varicose veins is from 41.2 to 62.8% among women and 13.9 to 37.9% among men.^1-3^

Traditional treatment of GSV insufficiency involves ligation at the saphenofemoral junction combined with partial or total stripping of the great saphenous vein. However, recent advances in endovascular techniques, such as ultrasound guided foam sclerotherapy (USGFS), endothermal radio frequency ablation (RFA) and endovenous laser ablation (ELA) have been widely adopted. These techniques are conducted under local anesthesia and have transformed management of chronic venous insufficiency (CVI), offering outpatient treatments with minimal recovery times and improved quality of life when compared to conventional surgery.^4-6^ As a result, the medical literature and recent guidelines recommend endovenous ablation, which includes both ELA and RFA, as the “gold standard” for treatment of GSV insufficiency.^7-9^

After conducting the GSV ablation procedure, it is common practice to apply compression to the treated limb, with the theoretical intention of reducing edema, ecchymosis, and pain, in addition to preventing superficial or deep thrombosis. However, the need for and the ideal duration of compression remain controversial subjects. Currently, in the context of endovenous thermal ablation, there are no guidelines that define definitive recommendations, because the available scientific evidence is insufficient.^8,10^

A systematic review encompassing analysis of five randomized clinical trials with a total of 734 patients examined the ideal duration of compression after endovenous ablation of varicose veins. However, the majority of the studies did not find significant differences in bruising, time to recovery, or leg swelling.^11^ These findings suggest a lack of solid evidence in favor of prolonged use of compression after endovenous ablation of varicose veins.

Indeed, the efficacy of compression therapy after endovenous treatment has been questioned recently. Ayo et al.^12^ compared compression for 7 days with no compression and did not find any differences in clinical measures (vein closure) or in patient-reported outcomes (pain, ecchymosis, and quality of life) after endovenous ablation.

Despite its widespread adoption in clinical practice, the benefits of compression therapy after endovenous treatment have recently been the subject of debate. Prescription of compression stockings after interventions for treatment of varicose veins is a common practice.

The objective of this study was to identify the Brazilian Society of Angiology and Vascular Surgery (SBACV) members’ current compression therapy practices after ablation of the GSV and use this information to guide future research into use of compression in this context. By obtaining relevant data from society members, this study has the potential to make significant contributions to understanding and optimization of this essential clinical practice.

## METHODS

This was a descriptive, cross-sectional study and the research protocol was approved by the Institutional Research Ethics Committee at the Hospital Municipal São José in Joinville, Santa Catarina, Brazil, under decision number 3.563.637 (Ethics Appraisal Submission Certificate: 17600619.90000.5362). All of the participants agreed to sign a free and informed consent form and the study complied with all of the ethical principles set out in Resolution 466/12 and the ethical standards contained in the Good Publication Practice Guidelines, developed by the Committee on Publication Ethics (COPE).^13^

A structured questionnaire on thermal ablation of the GSV and post-endovenous ablation compression comprising 14 questions and closed, multiple-choice response options, was developed by the lead author and reviewed by a group of vascular surgeons (Table 1). The document was adapted for Google Forms^®^, which was used to collect the responses. From September to November of 2021, a link to the survey was sent to Brazilian angiologists and vascular surgeons in online groups for discussion of clinical cases and text messaging software such as WhatsApp*®,* or via e-mail. The questionnaire was sent with an accompanying invitation letter explaining the study objectives.

**Table 1:** Items included in the source questionnaire used to obtain the study results.

Items on the questionnaire	
1.	*Input your CRM and State*
2.	*Do you perform thermal ablation of the great saphenous vein?*
	Yes Ž
	No Ž
3.	*What technique do you use for thermal ablation of the great saphenous vein?*
	Laser Ž
	Radio frequency Ž
4.	*Where do you usually perform thermal ablation of the great saphenous vein?*
	Hospital Ž
	Day hospital Ž
	Office Ž
5.	*Which anesthetic technique do you usually use for thermal ablation of the great saphenous vein?*
	General anesthesia Ž
	Epidural Ž
	Spinal anesthesia Ž
	Peripheral nerve blockŽ
	Local anesthesia with sedation Ž
	Local anesthesia without sedation Ž
6.	*Do you use tumescence?*

	Yes Ž
	No Ž
7.	*Do you perform thermal ablation of the great saphenous vein below the knee?*
	Yes Ž
	No Ž
	If necessary, at a separate time Ž
8.	*Do you treat associated varicose veins during the same procedure as thermal ablation of the great saphenous vein?*
	Yes Ž
	No Ž
9.	*Which technique do you use to treat associated varicose veins during the same procedure as thermal ablation of the great saphenous vein?*
	Phlebectomy Ž
	Foam Ž
	I don’t treat associated varicose veins during the same procedure as ablation da saphenous vein Ž
10.	*What type of compression do you use immediately after thermal ablation of the great saphenous vein?*
	Crepe bandages Ž
	Elastic bandages Ž
	Inelastic bandages Ž
	18-23 mmHg compression stockings Ž
	20-30 mmHg compression stockings Ž
	35 mmHg compression stockings Ž
	30-40 mmHg compression stockings Ž
	Extrinsic compression alone Ž
	Extrinsic compression combined with compression stockings Ž
	A different type of compression Ž
	I do not use compression immediately after ablation of the saphenous vein Ž
11.	*How long do you maintain this initial compression?*
	1 day Ž
	2 days Ž
	3 days Ž
	4 days Ž
	5 days Ž
	6 days Ž
	7 days Ž
	10 days Ž
	15 days Ž
	Outros Ž
	I do not use compression immediately after ablation of the saphenous vein Ž
12.	*Do you use any other type of compression after the initial compression?*
	Yes Ž
	No Ž
13.	*What type of additional compression do you usually use?*
	18-23 mmHg compression stockings Ž
	20-30 mmHg compression stockings Ž
	30-40 mmHg compression stockings Ž
	Other types of additional compression Ž
	I do not use any additional type of compression Ž
14.	How long do you maintain this additional compression?
	7 days Ž
	30 days (4 weeks) Ž
	6 weeks Ž
	60 days (8 weeks) Ž
	90 days (12 weeks Ž
	Other duration Ž
	I do not use any additional type of compression Ž

Source: the authors, 2023.

The first step in the study was presentation of the free and informed consent form, which was shown to participants before they completed the questionnaire. The respondents were directed to the free and informed consent form via a link, which led to an required field icon with the declaration: “I have read and agree to the terms of the free and informed consent form”. By clicking this button, the participant agreed to the terms of the study and this consent was recorded in the database as proof of participation in and acceptance of the study.

The following strategies were adopted to apply exclusion criteria. The first item of the questionnaire was “Input your CRM and State” (a Brazilian physician’s CRM is their state medical board registration number). It was thus possible to exclude duplicate questionnaires, respondents whose names did not match the CRM registers, and respondents who were not members of the SBACV. The second item was also an exclusion strategy, since the question was “Do you perform thermoablation of the great saphenous vein?”, making it possible to exclude questionnaires from respondents who stated they did not perform thermal ablation of the GSV, since this study is designed to analyze compression therapy after thermal ablation of the GSV.

For the purposes of sample size calculation, the SBACV had 3,551 members at the time of data collection. It was estimated that 30% of these members perform endovenous ablation of the GSV and that approximately 30% of this percentage would respond to the questionnaire. Therefore, the calculation of the sample necessary to enable statistical inference is around 320 questionnaires responded by members, considering a 4% maximum margin of error and a 95% confidence level.

For analyses, the dataset was stored in a Microsoft Excel^®^ spreadsheet and imported into IBM’s Statistical Package for the Social Sciences, version 23 (International Business Machines, NY, United States). The normality of data was verified using the Shapiro-Wilk test. Descriptive statistics were employed, with measures of central tendency and variability for numerical variables and absolute and relative frequencies for categorical variables.

## RESULTS

A total of 430 questionnaires were returned, 49 (11.4%) of which were excluded because the respondents were not members of the SBACV, 18 (4.2%) because they were duplicates, and one (0.2%) because the professional concerned responded to question two that they did not perform thermal ablation of the great saphenous vein. Therefore, the dataset for the study comprised 362 (84.2%) questionnaires.

With relation to the type of thermal ablation employed, the majority of participants (73.5%) reported using the ELA technique for GSV ablation. Spinal anesthesia was employed for procedures by 237 (65.5%) professionals and 213 (58.8%) of these conducted ablation in a hospital environment (Table 2).

**Table 2:** Type of thermal ablation used, anesthetic technique used, and setting where thermal GSV ablation is performed.

Variables	n (%)
*Technique used for ablation*	
Laser	266 (73.5)
Radio frequency	96 (26.5)
Anesthetic technique used	
Spinal anesthesia	237 (65.5)
Epidural	27 (7.5)
Local anesthesia with sedation	59 (16.3)
Local anesthesia without sedation	23 (6.3)
General anesthesia	8 (2.2)
Peripheral nerve block	3 (0.8)
Other	5 (1.4)
*GSV ablation setting*	
Hospital	213 (58.8)
Day hospital	126 (34.8)
Office	22 (6.1)
Other a	1 (0.3)

Source: the authors, 2023. GSV= great saphenous vein. Note: values expressed as absolute (n) and relative (%) frequencies.

a the participant who chose ‘other’ did not define the setting.

Table 3 shows the results for findings related to the surgical techniques employed. A majority of the participants (95.0%) stated they provoked tumescence during GSV ablation. Additionally, 181 (50.0%) respondents performed ablation below the knee and 343 (94.8%) treated varicose veins during the same procedure as GSV ablation. Phlebectomy was the technique most often employed to treat associated varicose veins, employed by 332 (91.7%) professionals.

**Table 3:** Surgical techniques employed for thermal ablation of the great saphenous vein.

Variables	n (%)
*Tumescence*	
No	18 (5.0)
Yes	344 (95.0)
*Ablation below the knee*	
No	156 (43.1)
Yes	181 (50.0)
If necessary, at a different time	7 (1.9)
Other	18 (5.0)
*Treatment of varicose veins concurrently with ablation*	
No	19 (5.2)
Yes	343 (94.8)
*Technique used to treatment of associated varicose veins concurrently with ablation*	
Phlebectomy	332 (91.7)
Foam	11 (3.0)
Does not treat varicose veins concurrently with ablation	19 (5.2)

Source: the authors, 2023. Note: values expressed as absolute (n) and relative frequencies (%).

**Figure 1:**
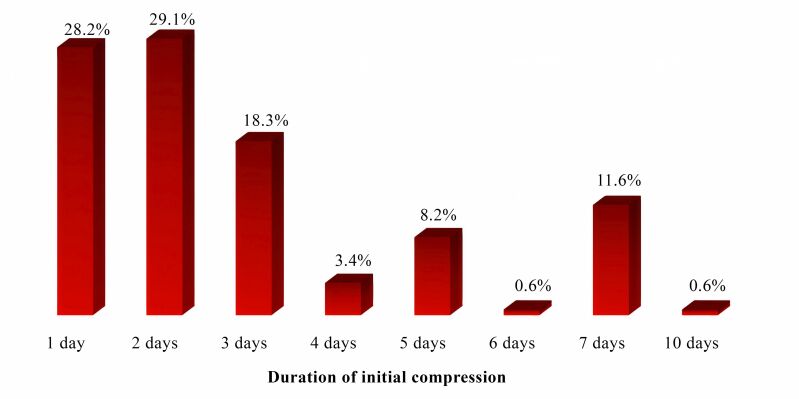
Duration, in days, of initial compression prescribed by study participants after thermoablation of the great saphenous vein. Source: the authors, 2023.

With relation to the type of initial compression prescribed for patients who underwent ablation of the GSV, the largest group of professionals (34.3%) stated that they indicated 35 mmHg compression stockings, followed by crepe bandages, recommended by 94 (26.0%) of the vascular surgeons who participated in the study (Table 4). The minimum duration reported was 1 day and the maximum was 10 days, with a median of 2 (1 – 3) days (Figure 1).

**Table 4:** Types of initial compression the study participants recommend after thermoablation of the great saphenous vein.

Type of compression	n (%)
Crepe bandages	94 (26.0)
Inelastic bandages	7 (1.9)
Elastic bandages	10 (2.8)
Extrinsic compression combined with compression stockings	52 (14.4)
Extrinsic compression alone	3.0 (0.8)
18-23 mmHg compression stockings	8 (2.2)
20-30 mmHg compression stockings	45 (12.4)
35 mmHg compression stockings	124 (34.3)
30-40 mmHg compression stockings	9 (2.5)
I don’t use compression immediately after GSV ablation	3 (0.8)
Other type of compression	7 (1.9)

Source: the authors, 2023. GSV= great saphenous vein. Note: values expressed as absolute (n) and relative (%) frequencies.

When asked about additional compression, 320 (88.4%) professionals recommended some type of additional compression after the initial compression method, whereas 42 (11.6%) vascular surgeons stated that they did not prescribe any type of additional compression. Among those who did use additional compression after the initial method, 20-30 mmHg compression stockings were the most common type, prescribed by 293 (80.9%) professionals, followed by 18-23 mmHg compression stockings, prescribed by 14 (3.9%) respondents. Additionally, 12 (3.3%) vascular surgeons used 30-40 mmHg compression stockings as additional compression method after GSV ablation and one (0.3%) participant reported prescribing a different type of compression (Table 5).

**Table 5:** Types of additional compression the study participants recommend after thermoablation of the great saphenous vein.

Type of compression	n (%)
18-23 mmHg compression stockings	14 (3.9)
20-30 mmHg compression stockings	293 (80.9)
30-40 mmHg compression stockings	12 (3.3)
No additional compression	42 (11.6)
Other type of compression	1 (0.3)

Source: the authors, 2023. Note: values expressed as absolute (n) and relative (%) frequencies.

**Figure 2:**
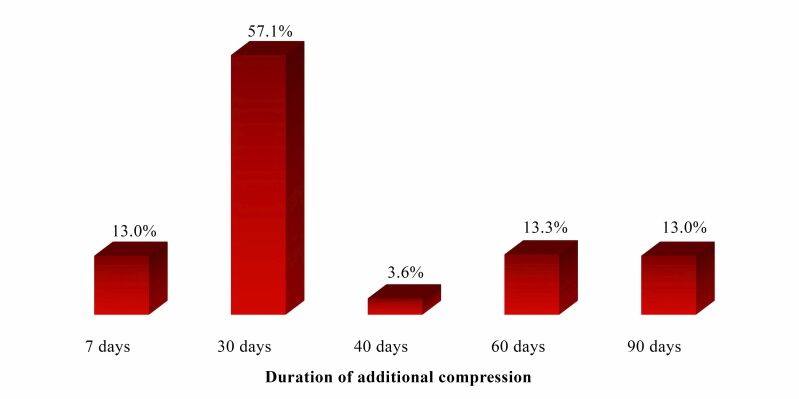
Duration, in days, of additional compression prescribed by study participants after thermoablation of the great saphenous vein. Source: the authors, 2023.

With relation to the duration of additional compression, the minimum duration reported was 7 days and the maximum was 90 days, with a median of 30 (30 – 60) days (Figure 2).

## DISCUSSION

Prescription of compression therapy after interventions to treat varicose veins is a practice that is adopted widely, although the need for it and the ideal duration of compression are still the subject of debate in the medical literature. To date, there is still no consensus on the ideal type of compression or the length of time for which it should be used after procedures for thermoablation of the GSV. Nevertheless, it is pertinent to point out that the majority of surgeons continue to employ the practice. In a study conducted in France in 2004, 97.1% of surgeons prescribed some type of compression after surgical treatment of varicose veins.^14^ In a British survey that analyzed the compression regimen prescribed after endovenous GSV ablation, whether chemical or thermal, it was found that all of the surgeons surveyed prescribed some type of compression after the procedure.^15^ In our own study, we observed a high rate of use of compression, with 99.2% of participants adopting the practice after thermal GSV ablation.

A comparison of the British study of the compression regimen used after endovenous GSV ablation with our study reveals significant nuances in clinical practices. According to the study by El-Sheikha et al.,^15^ members of the Vascular Society of Great Britain and Ireland adopted compression therapy for an average of 10 days after the procedure, with a range of 2 days to 6 weeks. A combination of bandages and compression stockings was the preference of 71% of surgeons, while 18% of the professionals used bandages alone. Moreover, 10% of the surgeons opted for bandages in conjunction with external compression devices and compression stockings, and a 1% minority chose bandages and external compression devices alone. These data reveal four distinct approaches, highlighting a comparatively greater diversity of practice during the first days after GSV ablation in our study. While the British surgeons recommended transition from bandages to compression stockings after around 2 days, maintenance of this additional compression for approximately 5 days, in line with our study, diverges from the more prolonged practice adopted by the Brazilian vascular surgeons in our study setting.

It is of interest to note that the duration of compression therapy and the type of compression stocking used after thermal GSV ablation vary substantially in different clinical trials. For example, in a study conducted by Bakker et al.,^16^ patients were randomized into two groups. Patients in the first group were prescribed compression for a short period, wearing thigh-high compression stockings with a pressure rating of 35 mmHg for 48 hours, while the second group wore the same compression stockings for 7 days.

In a clinical trial conducted by Mii et al.,^17^ one group of patients wore 20 mmHg compression stockings for periods of 1 to 2 days, whereas the other group wore compression stockings for longer periods, of from 1 to 4 weeks.

The justification for applying compression after venous procedures is to establish adequate pressure in the treated veins, with the objective of preventing or minimizing possible complications, such as inflammation, pain, bruising, and bleeding and the risk of developing superficial or deep venous thrombosis.^18^ This practice is widely adopted to avoid these complications, although it is relevant to observe that the evidence to support its efficacy is still limited.

In order to guarantee effective occlusion of a traumatized vein, it is essential to apply adequate pressure. In a lying position, this equates to pressures exceeding 10-15 mmHg, while when in a vertical position, it is necessary to reach 40-50 mmHg.^19^ Inelastic bandaging can exceed 50 mmHg in the thigh region, but elastic stockings only apply low mmHg in the same area. The target can be achieved if the stockings are worn over an excentric compression support that is positioned directly over a treated vein or over the course of a vein that has been removed surgically.^20^ Therefore, the correct choice of compression after treatment for varicose veins is crucial, since the pressure exerted plays an essential role in prevention of complications and promotion of adequate patient recovery.

The efficacy of compression therapy after endovenous ablation has been widely debated, especially after clinical trials that have directly compared use of compression with its absence after endovenous thermal ablation of the GSV. Relevant studies^12,21,22^ did not identify significant differences in crucial outcomes such as rate of GSV occlusion, quality of life, clinical venous severity score (CVSS), patient satisfaction, or pain, irrespective of use of compression. These findings were corroborated by a meta-analysis of seven randomized controlled trials, which concluded that while compression stockings may slightly minimize postoperative pain, they did not significantly impact quality of life, incidence of complications, or time taken to return to work.^23^ This result suggests that compression stockings may not be necessary for patients with grade C2 to C3 varicose veins, considering the inconvenience and difficulties related to wearing them and patients’ compliance with their use. In a meta-analysis by Zhang et al.,^24^ which analyzed four randomized controlled trials with a total of 552 patients, a reduction in postoperative pain was also reported for those who used compression therapy compared to those who did not, although this advantage did not extend to other important indicators. This indicates that although it offers some relief from pain, compression therapy may not confer additional significant benefits.

Concomitant treatment of varicose veins can also be considered when deciding on use of compression after thermal GSV ablation procedures. The majority of published studies about use of compression after surgical procedures did not include patients treated concomitantly for associated varicose veins.^12,16,21,22,25^ A study by Bootun et al.^26^ that analyzed patients with GSV reflux who underwent thermoablation, with or without concomitant phlebectomies, randomized patients to receive compression stockings for 7 days or no type of compression. The group with compression had considerably reduced mean pain levels, particularly patients who underwent phlebectomy concomitantly with GSV treatment. In a recent randomized study, Coelho et al.^27^ observed that compression therapy for 7 days during the postoperative period after phlebectomy can prevent edema secondary to the procedure and, consequently, improve patient comfort. Notably, 94.8% of the Brazilian vascular surgeons who completed the questionnaire in our study treat varicose veins during the same procedure as the GSV treatment. Phlebectomy was the most widely used technique, employed by 91.7% of these professionals. These findings explain the use of compression stockings for prolonged periods, ensuring continuous improvement in clinical results and relief of post-procedural pain and edema.

This study stands out as the only one available that has investigated use of compression after endovascular treatment of the GSV in the Brazilian setting. Compared with similar international studies, it is notable for the significant quantity of the data published.

However, it is extremely important to recognize the limitations inherent to the study. The exact number of potential participants who received the electronic questionnaire can not be determined precisely because of its distribution by e-mail to SBACV members and via social networks, such as the Fórum Vascular® and WhatsApp®. Moreover, it is relevant to observe that the number of participants interviewed in this study is relatively small compared to the universe of vascular surgeons in Brazil. Another limitation of the study was not investigating use of compression therapy at different CEAP classification grades. Although studies indicate that patients with chronic venous insufficiency (CVI) at the initial stages (C2 and C3) may not benefit from compression stockings after procedures, the applicability of these results at more advanced stages (C5 and C6) remains uncertain. ^23^ Moreover, it is important to emphasize the need to investigate patients’ compliance with compression therapy, which is a factor that we recognize as a limitation of the present study. Many patients report discomfort with prolonged compression therapy, especially in tropical climates, such as in Brazil, as shown by Biswas et al.,^18^ who found that more than 60% of patients stopped wearing elastic stockings before the end of treatment. These limitations show the need for further research to optimize compression practices and improve care for patients with varicose veins.

To the extent that we have explored the Brazilian vascular surgeons’ practices and approaches in this initial survey, the relevance of these data as a solid foundation to support guidelines related to compression therapy after treatment of venous insufficiency is evident. Our study is compatible with previous publications, demonstrating widespread adoption of compression therapy after vascular procedures. These discoveries offer a valuable perspective for development of health care policies, underscoring the need for clear and evidence-based guidelines for optimization of the quality of care for patients who undergo GSV ablation and related vascular procedures.

## CONCLUSIONS

Based on the data presented and the information provided, it can be concluded that compression therapy is widely employed by Brazilian vascular surgeons after GSV ablation. The study revealed a diverse range of compression types, reflecting the differing preferences in the professionals’ therapeutic choices. A wide variety was observed in the compression devices employed after the initial post GSV thermal ablation period. After this initial phase, the majority of professionals chose to prescribe additional compression therapy, the most common choice being compression stockings with a pressure range of 20-30 mmHg. The variability of compression therapy approaches after GSV ablation underscores the need for clearer, evidence-based guidelines for health professionals, in order to ensure the quality and efficacy of the care provided to the patients who undergo these procedures.
