# Supplementary material for: Post-saphenous vein ablation compression therapy practices: a study among members of the Brazilian society of angiology and vascular surgery
Source: J Vasc Bras. 2025 Feb 14;24:e20230182. doi: 10.1590/1677-5449.202301822 (PMC11841618; doi:10.1590/1677-5449.202301822)
Supplement: Translation

# Translation: Práticas de terapia compressiva pós-ablação da veia safena magna: um estudo entre os membros da Sociedade Brasileira de Angiologia e Cirurgia Vascular

**DOI:** 10.1590/1677-5449.202301821

**Authors:** Fabricio Duarte, Flavia Del Castanhel, Marcondes Antônio de Medeiros Figueiredo, Getúlio Rodrigues de Oliveira

**Affiliations:** 1 Universidade Federal de Santa Catarina – UFSC, Florianópolis, SC, Brasil.; 2 Centro Universitário IMEPAC, Araguari, MG, Brasil.

**Keywords:** insuficiência venosa, varizes, meias de compressão, bandagens compressivas, veia safena, ténicas de ablação

## Abstract

**Contexto:**

Apesar dos estudos, a definição do regime de compressão ideal após a ablação da veia safena magna por radiofrequência e por *endolaser* ainda é controversa.

**Objetivo:**

Identificar as práticas atuais de terapia compressiva após a ablação da veia safena magna entre os membros da Sociedade Brasileira de Angiologia e Cirurgia Vascular.

**Métodos:**

Um questionário eletrônico de múltipla escolha sobre a compressão pós-ablação endovenosa foi desenvolvido e divulgado on-line aos cirurgiões vasculares brasileiros por 60 dias.

**Resultados:**

Das 430 respostas obtidas, 362 (84,2%) foram consideradas válidas. A ablação a *laser* foi a técnica predominante (73,5%), sendo a sua maioria realizada em hospital ou hospital-dia. Além disso, 94% dos cirurgiões tratavam as varizes associadas no mesmo procedimento, sendo a flebectomia a técnica mais comumente empregada. Após a ablação da veia safena magna, 99% dos cirurgiões aplicaram compressão imediatamente; 34,3% indicaram o uso de meias de 35 mmHg; 26% deram preferência a ataduras de crepom; e 12,4% optaram pelo uso de meias de 20-30 mmHg, com média de uso de 2,79 (±2 dias). Após esse período, 88,4% utilizaram compressão adicional, com meias de 20-30 mmHg (80,9%), com média de uso de 39,3 (±24,0 dias).

**Conclusões:**

A terapia compressiva é amplamente adotada após a ablação térmica da veia safena magna. Observa-se diversidade na prática imediatamente após a ablação, mas após uma fase inicial, a maioria dos cirurgiões opta por compressão adicional com predominância de meias de 20-30 mmHg.

## INTRODUÇÃO

A insuficiência da veia safena magna (VSM) é a causa mais comum de insuficiência venosa crônica. No Brasil, a prevalência de varizes nos membros inferiores é de 41,2 a 62,8% nas mulheres e de 13,9 a 37,9% nos homens^1-3^ .

Tradicionalmente, o tratamento da insuficiência da VSM envolvia a ligadura da junção safeno-femoral associada à safenectomia magna, seja parcial ou total. No entanto, avanços recentes nas técnicas endovasculares, como a escleroterapia com espuma guiada por ultrassom (EEGU), a ablação endotérmica com radiofrequência (RFA) e a ablação a *laser* endovenoso (LE) têm sido amplamente adotadas. Essas técnicas, realizadas com anestesia local, transformaram o manejo da insuficiência venosa crônica (IVC), oferecendo tratamento ambulatorial com tempo mínimo de recuperação e melhorias na qualidade de vida comparáveis à cirurgia convencional^4-6^ . Como resultado, a literatura médica e as diretrizes recentes recomendam a ablação endovenosa, que inclui o LE ou a RFA, como o “padrão-ouro” no tratamento da insuficiência da VSM^7-9^ .

Após a realização do procedimento de ablação da VSM, é uma prática comum o uso de compressão no membro tratado, com a intenção teórica de reduzir edema, equimoses e dor, além de prevenir trombose superficial ou profunda. No entanto, a necessidade e a duração ideais da compressão continuam sendo temas controversos. Atualmente, no contexto da ablação térmica endovenosa, não existem diretrizes que estabeleçam uma recomendação definitiva devido à insuficiência de evidências científicas disponíveis^8,10^ .

Uma revisão sistemática abrangendo a análise de cinco ensaios clínicos randomizados com um total de 734 pacientes, examinou a duração ideal da terapia de compressão após a ablação endovenosa de varizes. Entretanto, a maioria desses estudos não encontrou diferenças significativas em relação a hematomas, tempo de recuperação e inchaço na perna^11^ . Esses achados sugerem a falta de evidências sólidas a favor da utilização prolongada da compressão após a ablação endovenosa de varizes.

De fato, a eficácia da terapia de compressão após o tratamento endovenoso tem sido objeto de questionamento recentemente. Ayo et al.^12^ compararam a compressão por 7 dias com a ausência de compressão e não encontraram diferenças nas medidas clínicas (obliteração da veia) e nos desfechos relatados pelos pacientes (dor, equimose e qualidade de vida) após a ablação endovenosa.

Apesar de sua ampla adoção na prática clínica, os benefícios da terapia de compressão após tratamento endovenoso recentemente têm sido alvo de debate. A prescrição de meias de compressão após intervenções para o tratamento de varizes é uma prática comum.

Este estudo teve como objetivo identificar a prática atual da terapia compressiva após ablação térmica da VSM dos membros da Sociedade Brasileira de Angiologia e Cirurgia Vascular (SBACV) e utilizar essas informações para direcionar futuras pesquisas sobre o uso da compressão nesse contexto. O estudo, ao obter dados relevantes junto a essa sociedade, tem o potencial de contribuir significativamente para a compreensão e o aprimoramento dessa prática clínica fundamental.

## MÉTODOS

Este foi um estudo transversal e descritivo, cujo projeto de pesquisa foi aprovado pelo Comitê de Ética em Pesquisa Institucional do Hospital Municipal São José, Joinville, Santa Catarina (Certificado de Apresentação de Apreciação Ética: 17600619.90000.5362), com número do parecer 3.563.637. Todos os participantes envolvidos concordaram com o Termo de Consentimento Livre e Esclarecido e o estudo seguiu todos os preceitos éticos considerados na Resolução 466/12, bem como as normas éticas contidas no Good Publication Practice Guidelines, documento elaborado por The Committee on Publication Ethics (COPE)^13^ .

Um questionário estruturado, contendo 14 perguntas com escala de respostas fechadas tipo múltipla escolha, relacionados à ablação térmica da VSM e à compressão pós-ablação endovenosa, foi desenvolvido pelo autor principal deste trabalho e revisado por um grupo de cirurgiões vasculares (Tabela 1). Esse documento foi adaptado ao Google Forms^®^, ferramenta utilizada para obtenção das respostas. No período de setembro a novembro de 2021, o *link* da pesquisa foi divulgado entre os angiologistas e cirurgiões vasculares brasileiros através de grupos de discussão de casos clínicos em *softwares* de mensagens de texto como Whatsapp*®,* ou através de *e-mail*. Junto ao questionário, foi enviada uma carta-convite explicando os objetivos do estudo.

**Tabela 1:** Itens que compuseram o questionário fonte para obtenção dos resultados do estudo.

**Itens do questionário**	
1.	*Insira seu CRM e Unidade da Federação*
2.	*Você faz a termoablação da veia safena magna?*
	Sim Ž
	Não Ž
3.	*Qual técnica você usa para fazer a termoablação da veia safena magna?*
	*Laser* Ž
	Radiofrequência Ž
4.	*Onde você costuma realizar a termoablação da veia safena magna?*
	Hospital Ž
	Hospital-dia Ž
	Consultório Ž
5.	*Qual técnica anestésica você geralmente usa para realizar a termoablação da veia safena magna?*
	Anestesia geral Ž
	Peridural Ž
	Raquianestesia Ž
	Bloqueio de nervo periférico Ž
	Anestesia local com sedação Ž
	Anestesia local sem sedação Ž
6.	*Você faz tumescência?*

	Sim Ž
	Não Ž
7.	*Você faz a termoablação da veia safena magna abaixo do joelho?*
	Sim Ž
	Não Ž
	Se necessário, em um segundo tempo Ž
8.	*Você trata as varizes associadas no mesmo momento da termoablação da veia safena magna?*
	Sim Ž
	Não Ž
9.	*Qual técnica você usa para tratar as varizes associadas no mesmo momento da termoablação da veia safena magna?*
	Flebectomia Ž
	Espuma Ž
	Não trato as varizes associadas no mesmo momento da ablação da veia safena Ž
10.	*Qual tipo de compressão você usa imediatamente após a termoablação da veia safena magna?*
	Atadura de crepom Ž
	Atadura elástica Ž
	Atadura inelástica Ž
	Meia de compressão 18-23 mmHg Ž
	Meia de compressão 20-30 mmHg Ž
	Meia de compressão 35 mmHg Ž
	Meia de compressão 30-40 mmHg Ž
	Compressão extrínseca isolada Ž
	Compressão extrínseca associada a meia de compressão Ž
	Outro tipo de compressão Ž
	Não uso compressão imediatamente após a ablação da veia safena Ž
11.	Por q*uanto tempo você deixa esta compressão inicial?*
	1 1 dia Ž
	2 2 dias Ž
	3 3 dias Ž
	4 4 dias Ž
	5 5 dias Ž
	6 6 dias Ž
	7 7 dias Ž
	10 dias Ž
	15 dias Ž
	Outros Ž
	Não uso compressão imediatamente após a ablação da veia safena Ž
12.	*Após a compressão inicial, você utiliza algum outro tipo de compressão?*
	Sim Ž
	Não Ž
13.	*Qual tipo de compressão adicional você geralmente usa?*
	Meia de compressão 18-23 mmHg Ž
	Meia de compressão 20-30 mmHg Ž
	Meia de compressão 30-40 mmHg Ž
	Outros tipos de compressão adicional Ž
	Não associo nenhum tipo de compressão adicional Ž
14.	Por quanto tempo você deixa essa compressão adicional?
	7 dias Ž
	30 dias (4 semanas) Ž
	6 semanas Ž
	60 dias (8 semanas) Ž
	90 dias (12 semanas Ž
	Outros Ž
	Não associo nenhum tipo de compressão adicional Ž

Fonte: elaborado pelos autores, 2023.

O primeiro passo da pesquisa envolve a introdução do Termo de Consentimento Livre e Esclarecido (TCLE), o qual foi apresentado aos participantes antes de preencherem o questionário. Os respondentes eram direcionados ao TCLE através de um *link*, onde encontravam um ícone de preenchimento obrigatório com a declaração: “Li e concordo com os termos do TCLE”. Ao clicar nesse botão, o participante concordava com os termos da pesquisa e esse consentimento era registrado no banco de dados como prova de sua participação e aceitação da pesquisa.

Como critério de exclusão, adotaram-se as seguintes estratégias: o primeiro item do questionário foi “Insira seu CRM e Unidade da Federação”. Assim, foi possível excluir questionários em duplicidade, aqueles cujos nomes não correspondiam ao CRM cadastrado, bem como os que os respondentes não eram membros da SBACV. Outra estratégia foi o segundo item, em que a pergunta foi “Você faz termoablação da veia safena magna?”. Dessa forma, também foi possível excluir questionários dos respondentes que citaram que não realizam a termoablação da VSM, visto que este estudo se propôs a analisar a terapia compressiva após a ablação térmica da VSM.

Para fins de cálculo amostral, a SBACV era composta, no momento da coleta de dados, por 3.551 membros. Estimava-se que 30% dos seus participantes realizavam a ablação endovenosa da VSM e que aproximadamente 30% desse percentual responderia ao questionário. Dessa forma, o cálculo da amostra necessária para permitir inferência estatística está em torno de 320 questionários respondidos pelos associados, considerando-se uma margem de erro máxima de 4% e nível de confiança de 95%.

Para executar as análises, o conjunto de dados foi armazenado em planilha Microsoft Excel^®^ e importado para o programa IBM Statistical Package for the Social Science (International Business Machines, NY, EUA) versão 23. A normalidade dos dados foi verificada por meio do teste de Shapiro-Wilk. A estatística foi descritiva, com medidas de tendência central e variabilidade para as variáveis numéricas, enquanto as categóricas foram por meio de frequência absoluta e relativa.

## RESULTADOS

Foram respondidos 430 questionários, dos quais 49 (11,4%) foram excluídos por identificarem que os respondentes não faziam parte da SBACV, 18 (4,2%) por duplicidade nas respostas e um (0,2%) porque o profissional respondeu na segunda pergunta do questionário que não fazia termoablação da veia safena magna. Portanto, 362 (84,2%) questionários formaram o conjunto de dados do estudo.

Em relação ao tipo de ablação térmica utilizada, a maioria dos participantes (73,5%) relatou fazer uso da técnica LE para ablação da VSM. A raquianestesia foi a técnica anestésica utilizada por 237 (65,5%) profissionais para realizar o procedimento, sendo executada em ambiente hospitalar por 213 (58,8%) desses (Tabela 2).

**Tabela 2:** Tipo de ablação térmica utilizada, técnica anestésica usada e local onde a termoablação da VSM é realizada.

Variáveis	n (%)
*Técnica utilizada para executar a ablação*	
*Laser*	266 (73,5)
Radiofrequência	96 (26,5)
*Técnica anestésica empregada*	
Raquianestesia	237 (65,5)
Peridural	27 (7,5)
Anestesia local com sedação	59 (16,3)
Anestesia local sem sedação	23 (6,3)
Anestesia geral	8 (2,2)
Bloqueio de nervo periférico	3 (0,8)
Outro	5 (1,4)
*Local realização ablação VSM*	
Hospital	213 (58,8)
Hospital-dia	126 (34,8)
Consultório	22 (6,1)
Outro a	1 (0,3)

Fonte: elaborado pelos autores, 2023. VSM= veia safena magna. Nota: valores expressos em frequência absoluta (n) e relativa (%).

a participante não informou local ao optar por essa categoria de resposta.

A Tabela 3 mostra os resultados no que se refere aos achados acerca das técnicas cirúrgicas empregadas. A maioria dos participantes (95,0%) relatou fazer tumescência durante a ablação da VSM. Além disso, 181 (50,0%) dos respondentes faziam ablação abaixo do joelho e 343 (94,8%) tratavam varizes no mesmo momento em que executavam a ablação da VSM, sendo a flebectomia a técnica mais empregada para tratar as varizes associadas, utilizada por 332 (91,7%) desses profissionais.

**Tabela 3:** Técnicas cirúrgicas empregadas para executar a termoablação da veia safena magna.

Variáveis	n (%)
*Tumescência*	
Não	18 (5,0)
Sim	344 (95,0)
*Ablação abaixo do joelho*	
Não	156 (43,1)
Sim	181 (50,0)
Se necessário, realiza em segundo momento	7 (1,9)
Outro	18 (5,0)
*Tratamento de varizes concomitantemente à ablação*	
Não	19 (5,2)
Sim	343 (94,8)
*Técnica usada no tratamento de varizes associada à ablação*	
Flebectomia	332 (91,7)
Espuma	11 (3,0)
Não trata varizes junto com ablação	19 (5,2)

Fonte: elaborado pelos autores, 2023. Nota: valores expressos em frequência absoluta (n) e relativa (%).

Em relação ao tipo de compressão inicial prescrita aos pacientes submetidos à ablação da VSM, a maioria dos profissionais (34,3%) relatou que indicava a meia de compressão 35 mmHg, seguida da atadura de crepom, recomendada por 94 (26,0%) dos cirurgiões vasculares participantes no estudo (Tabela 4). O tempo mínimo registrado foi de 1 dia e o máximo foi de 10 dias, com mediana de 2 (1 – 3) dias (Figura 1).

**Tabela 4:** Tipos de compressão inicial recomendados pelos participantes do estudo após termoablação da veia safena magna.

Tipo de compressão	n (%)
Atadura de crepom	94 (26,0)
Atadura inelástica	7 (1,9)
Atadura elástica	10 (2,8)
Compressão extrínseca associada a meia de compressão	52 (14,4)
Compressão extrínseca isolada	3,0 (0,8)
Meia de compressão 18-23 mmHg	8 (2,2)
Meia de compressão 20-30 mmHg	45 (12,4)
Meia de compressão 35 mmHg	124 (34,3)
Meia de compressão 30-40 mmHg	9 (2,5)
Não uso compressão imediatamente após a ablação da VSM	3 (0,8)
Outro tipo de compressão	7 (1,9)

Fonte: elaborado pelos autores, 2023. VSM= veia safena magna. Nota: valores expressos em frequência absoluta (n) e relativa (%).

**Figura 1:** Tempo de compressão inicial em dias utilizado pelos participantes em seus pacientes após termoablação da veia safena magna. Fonte: elaborado pelos autores, 2023.

Questionados acerca de compressão adicional, 320 (88,4%) profissionais recomendavam alguma compressão adicional após utilização de uma inicial, enquanto 42 (11,6%) cirurgiões vasculares relataram não associar nenhum tipo de compressão adicional. Quanto aos que associavam compressão adicional após a inicial, a meia de compressão 20-30 mmHg foi a mais prescrita por 293 (80,9%) profissionais, seguida da meia de compressão 18-23 mmHg, prescrita por 14 (3,9%) dos respondentes. Além disso, 12 (3,3%) cirurgiões vasculares utilizam a meia de compressão 30-40 mmHg como método de compressão adicional após a ablação da VSM, e um (0,3%) participante relatou que prescrevia outro tipo de compressão (Tabela 5).

**Tabela 5:** Tipos de compressão adicional recomendados pelos participantes do estudo após termoablação da veia safena magna.

Tipo de compressão	n (%)
Meia de compressão 18-23 mmHg	14 (3,9)
Meia de compressão 20-30 mmHg	293 (80,9)
Meia de compressão 30-40 mmHg	12 (3,3)
Não associo nenhum tipo de compressão adicional	42 (11,6)
Outro tipo de compressão	1 (0,3)

Fonte: elaborado pelos autores, 2023. Nota: valores expressos em frequência absoluta (n) e relativa (%).

Em relação ao tempo de utilização da compressão adicional, o tempo mínimo registrado foi de 7 dias e o máximo de 90 dias, com uma mediana de 30 (30 – 60) dias (Figura 2).

**Figura 2:** Tempo de compressão adicional, em dias, utilizado pelos participantes em seus pacientes após termoablação da veia safena magna. Fonte: elaborado pelos autores, 2023.

## DISCUSSÃO

A prescrição de terapia compressiva após intervenções para o tratamento de varizes é uma prática amplamente adotada, embora a necessidade e a duração ideal da compressão permaneçam tópicos de debate na literatura médica. Até os dias atuais, não há um consenso sobre o tipo de compressão ideal e o tempo que essa deve ser usada após os procedimentos termoablativos da VSM. No entanto, é relevante destacar que a maioria dos cirurgiões continua a adotar essa prática. Em um estudo realizado na França, em 2004, 97,1% dos cirurgiões prescreveram algum tipo de compressão após o tratamento cirúrgico de varizes^14^ . Em uma enquete britânica que analisou o regime de compressão após ablação endovenosa da VSM, seja química ou térmica, foi constatado que todos os cirurgiões pesquisados prescreviam algum tipo de compressão após o procedimento^15^ . Em nosso próprio estudo, observamos uma alta taxa de utilização de compressão, com 99,2% dos participantes adotando essa prática após a ablação térmica da VSM.

A comparação entre a pesquisa britânica sobre o regime de compressão após a ablação endovenosa da VSM e nosso estudo revela nuances significativas nas práticas clínicas. No estudo conduzido por El-Sheikha et al.^15^ , membros da Sociedade Vascular da Grã-Bretanha e Irlanda adotaram, em média, uma terapia de compressão por 10 dias, com variação de 2 dias a 6 semanas após o procedimento. A combinação de ataduras e meias de compressão foi a preferência de 71% dos cirurgiões, enquanto apenas ataduras foram utilizadas por 18% dos profissionais. Além disso, 10% dos cirurgiões optaram por ataduras em conjunto com dispositivos de compressão externa e meias de compressão, e uma minoria de 1% escolheu ataduras e dispositivos de compressão externa isoladamente. Esses dados revelam quatro práticas distintas, destacando uma diversidade de abordagens comparativamente maior em nossa pesquisa nos primeiros dias pós-ablação da VSM. Embora os cirurgiões britânicos tenham recomendado a transição das bandagens para meias de compressão após cerca de 2 dias, em alinhamento com nosso estudo, a persistência dessa compressão adicional por aproximadamente 5 dias foi divergente da prática mais prolongada adotada pelos cirurgiões vasculares brasileiros em nosso contexto de pesquisa.

É interessante observar que a duração da terapia de compressão e o tipo de meia de compressão utilizado após a ablação térmica da VSM variam substancialmente em diferentes ensaios clínicos. Por exemplo, no estudo conduzido por Bakker et al.^16^ , os pacientes foram randomizados em dois grupos. O primeiro grupo usou compressão por um curto período, utilizando meias de compressão 7/8 com uma pressão de 35 mmHg por 48 horas, enquanto o segundo grupo usou as mesmas meias de compressão por 7 dias.

No ensaio clínico conduzido por Mii et al.^17^ , um grupo de pacientes utilizou meias de compressão de 20 mmHg por um período de 1 a 2 dias, enquanto o outro grupo utilizou as meias de compressão por um período mais prolongado de 1 a 4 semanas.

A justificativa para a aplicação da compressão após procedimentos venosos é estabelecer uma pressão adequada nas veias tratadas, com o objetivo de prevenir ou minimizar possíveis complicações, como inflamação, dor, hematomas, sangramento e o risco de desenvolver trombose venosa superficial ou profunda^18^ . Essa prática é amplamente adotada para evitar tais complicações, embora seja relevante observar que as evidências que sustentam sua eficácia ainda são limitadas.

Para garantir uma oclusão eficaz de uma veia traumatizada, é essencial aplicar a pressão adequada. Em posição deitada, isso significa superar os 10-15 mmHg, enquanto em posição vertical, é necessário alcançar 40-50 mmHg^19^ . Faixas inelásticas conseguem exceder 50 mmHg na região da coxa, mas meias elásticas fornecem apenas alguns mmHg nessa mesma área. Essa meta torna-se alcançável quando as meias são aplicadas sobre um suporte de compressão excêntrico, posicionado diretamente sobre a veia tratada ou sobre o trajeto da veia removida cirurgicamente^20^ . Assim, a escolha da compressão adequada após o tratamento de varizes é crucial, pois a pressão exercida desempenha um papel fundamental na prevenção de complicações e na promoção da recuperação adequada dos pacientes.

A eficácia da terapia compressiva pós-ablação endovenosa tem sido amplamente debatida, especialmente após ensaios clínicos que compararam diretamente o uso de compressão com a sua ausência após a ablação térmica endovenosa da VSM. Estudos relevantes^12,21,22^ não identificaram diferenças significativas em desfechos cruciais como taxa de oclusão da VSM, qualidade de vida, escore clínico de gravidade venosa (VCSS), satisfação do paciente e dor, independentemente do uso de compressão. Esses achados são reforçados por uma metanálise envolvendo sete ensaios controlados randomizados, que concluiu que as meias de compressão, embora possam minimizar levemente a dor pós-operatória, não impactam significativamente a qualidade de vida, a incidência de complicações ou o tempo de retorno ao trabalho^23^ . Esse resultado sugere que as meias de compressão podem não ser necessárias para pacientes com varizes de graus C2 a C3, considerando os inconvenientes e dificuldades associados à sua utilização e sua aderência entre os pacientes. Na metanálise descrita por Zhang et al.^24^ , que avaliou quatro ensaios controlados randomizados com um total de 552 pacientes, se indicou também uma redução na dor pós-operatória para aqueles que utilizaram a terapia de compressão em comparação com os que não utilizaram, embora essa vantagem não se estenda a outros indicadores importantes. Isso indica que, apesar de proporcionar algum alívio na dor, a terapia de compressão pode não trazer benefícios adicionais significativos.

O tratamento concomitante das varizes pode ser considerado ao decidir sobre o uso da compressão pós-procedimento de ablação térmica da VSM. A maioria dos estudos publicados sobre o uso da compressão após o procedimento cirúrgico não inclui tratamentos concomitantes para as varizes associadas^12,16,21,22,25^ . No estudo conduzido por Bootun et al.^26^ , que analisou pacientes com refluxo na VSM submetidos à termoablação, com ou sem flebectomias concomitantes, os pacientes foram randomizados para receber meias de compressão durante 7 dias ou nenhum tipo de compressão. O grupo com compressão associada teve níveis médios de dor consideravelmente reduzidos, principalmente os pacientes submetidos à flebectomia concomitantemente ao tratamento da VSM. Em um estudo randomizado recente, Coelho et al.^27^ observaram que a terapia de compressão de 7 dias no período pós-operatório após a flebectomia pode prevenir o edema secundário ao procedimento e, consequentemente, melhorar o conforto do paciente. Notavelmente, em nosso estudo, 94,8% dos cirurgiões vasculares brasileiros que responderam ao questionário tratam as varizes de forma concomitante ao tratamento da VSM, sendo a flebectomia a técnica mais amplamente utilizada, empregada por 91,7% desses profissionais. Esses achados fornecem justificativa para o uso de meias de compressão por um período prolongado, assegurando assim uma melhoria contínua nos resultados clínicos e alívio da dor e do edema pós-procedimento.

Este estudo se destaca por ser a única pesquisa disponível que aborda o uso da compressão após o tratamento endovascular da VSM no contexto brasileiro. Em comparação com estudos internacionais semelhantes, este é notável pela quantidade significativa de dados que foram publicados.

Contudo, é de extrema importância reconhecer as limitações inerentes a esta pesquisa. A quantidade exata de participantes que receberam o questionário eletrônico não pôde ser precisamente determinada devido à sua distribuição via *e-mail* entre os membros da SBACV e à sua divulgação em redes sociais, como o Fórum Vascular® e o WhatsApp®. Além disso, é relevante observar que o número de participantes entrevistados neste estudo é relativamente pequeno quando comparado ao universo de cirurgiões vasculares no Brasil. Outra limitação do estudo foi não investigar o uso da terapia compressiva em diferentes estágios da classificação CEAP. Embora estudos indiquem que pacientes com insuficiência venosa crônica (IVC) em estágios iniciais (C2 e C3) possam não se beneficiar de meias compressivas após procedimentos, a aplicabilidade desses resultados em estágios mais avançados (C5 e C6) permanece incerta^23^ . Além disso, é importante destacar a necessidade de investigar a adesão dos pacientes à terapia de compressão, um aspecto que reconhecemos como limitação do presente estudo. Muitos pacientes relatam desconforto com o uso prolongado da terapia de compressão, especialmente em climas tropicais como o do Brasil, como evidenciado por Biswas et al.^18^ , onde mais de 60% dos pacientes abandonaram o uso de meias elásticas antes do término da terapia. Essas limitações apontam para a necessidade de pesquisas futuras para aperfeiçoar as práticas de compressão e melhorar os cuidados aos pacientes com varizes.

À medida que exploramos as práticas e condutas dos cirurgiões vasculares brasileiros neste levantamento inicial, torna-se evidente a relevância desses dados como uma base sólida para orientar diretrizes relacionadas à terapia compressiva após o tratamento da insuficiência venosa. Nosso estudo está alinhado com publicações prévias, demonstrando a ampla adoção da terapia compressiva após os procedimentos vasculares. Essas descobertas oferecem uma perspectiva valiosa para o desenvolvimento de políticas de saúde, destacando a necessidade de diretrizes claras e baseadas em evidências para otimizar a qualidade do atendimento aos pacientes submetidos à ablação da VSM e procedimentos vasculares correlatos.

## CONCLUSÃO

Com base nos dados apresentados e nas informações fornecidas, é possível concluir que a terapia compressiva após a ablação da VSM é amplamente empregada pelos cirurgiões vasculares brasileiros. A pesquisa revelou uma diversidade de tipos de compressão utilizados, refletindo as diferentes preferências dos profissionais em suas escolhas terapêuticas. Observa-se uma ampla variedade de dispositivos de compressão em um período inicial após a ablação térmica da VSM. Após a fase inicial, a maioria dos profissionais optou por prescrever uma terapia de compressão adicional, sendo a meia de compressão com uma faixa de pressão de 20-30 mmHg a opção mais frequente. A variabilidade nas abordagens de terapia compressiva após a ablação da VSM destaca a necessidade de diretrizes mais claras e baseadas em evidências para orientar os profissionais de saúde, a fim de garantir a qualidade e eficácia dos cuidados prestados aos pacientes submetidos a esses procedimentos.
